# Undermining ribosomal RNA transcription in both the nucleolus and mitochondrion: an offbeat approach to target MYC-driven cancer

**DOI:** 10.18632/oncotarget.23579

**Published:** 2017-12-22

**Authors:** Stefano Rossetti, Andrzej J. Wierzbicki, Nicoletta Sacchi

**Affiliations:** ^1^ Department of Cancer Genetics and Genomics, Roswell Park Cancer Institute, Buffalo, NY 14263, USA

**Keywords:** Pol I-nucleolar rRNA transcription, POLRMT-mitochondrial rRNA transcription, MYC-driven proliferation, Pol I and POLRMT inhibitors

## Abstract

The MYC transcription factor coordinates, via different RNA polymerases, the transcription of both ribosomal RNA (rRNA) and protein genes necessary for nucleolar as well as mitochondrial ribogenesis. In this study we tested if MYC-coordination of rRNA transcription in the nucleolus and in the mitochondrion drives (cancer) cell proliferation. Here we show that the anti-proliferative effect of CX-5461, a Pol I inhibitor of rRNA transcription, in ovarian (cancer) cell contexts characterized by MYC overexpression is enhanced either by 2’-C-Methyl Adenosine (2’-C-MeA), a ribonucleoside that inhibits POLRMT mitochondrial rRNA (mt-rRNA) transcription and doxycycline, a tetracycline known to affect mitochondrial translation. Thus, hindering not only mt-rRNA transcription, but also mitoribosome function in MYC-overexpressing ovarian (cancer) cells, potentiates the antiproliferative effect of CX-5461. Targeting MYC-regulated rRNA transcription and ribogenesis in both the nucleolus and mitochondrion seems to be a novel approach worth of consideration for treating MYC-driven cancer.

## INTRODUCTION

The nucleolus is the major site of ribosomal RNA (rRNA) synthesis. Moreover, the mitochondrion is another site of rRNA synthesis. The MYC transcription factor regulates genes encoding proteins necessary for both nucleolar and mitochondrial rRNA transcription. MYC enables RNA Polymerase I (Pol I)-mediated nucleolar rRNA transcription by binding directly the rDNA promoter to induce chromatin modifications that enhance Pol I accessibility and facilitate the assembly of the Pol I transcription machinery [1–4]. MYC also controls RNA Polymerase II (Pol II)-mediated transcription of “Pol I regulon” genes encoding for basal components of the Pol I transcription machinery, including RRN3, UBTF, the SL1 complex component TAF1C, and the Pol I subunits POLR1B and POLR1E [5, 6]. In this way, MYC regulates the availability of nucleolar “Pol I regulon” factors necessary to activate rDNA genes [6]. As far as mitochondrial rRNA transcription is concerned, MYC regulates, via Pol II, the transcription of both the mitochondrial RNA polymerase POLRMT [7] and the mitochondrial transcription factor TFAM [8]. Both POLRMT and TFAM are necessary for transcription of mitochondrial DNA (mtDNA) into mitochondrial 12S and 16S rRNA (mt-rRNA) [9]. Moreover, MYC via Pol II controls the transcription of several proteins required for mitoribogenesis [10]. Hence the hypothesis that targeting both nucleolar and mitochondrial rRNA synthesis in MYC-overexpressing cancer can have a potential antiproliferative value.

In cancer of different histotypes MYC gene amplification or upregulation drive tumorigenesis by inducing stemness, promoting cell growth and proliferation, and hampering cell differentiation [11, 12]. Thus, drugs capable of inhibiting nucleolar Pol I-mediated rRNA transcription are emerging as new tools to target MYC-overexpressing cancer [13, 14]. One of these drugs is CX-5461. This small molecule, by selectively inhibiting Pol I-mediated rRNA transcription, can efficiently curb cancer cell proliferation both *in vitro* and *in vivo* [15–20]. Indeed, recent studies suggest that CX-5461 effectively targets MYC-driven proliferation in different cancer cell contexts, including lymphoma [16, 21], multiple myeloma [22], and prostate cancer [23, 24].

Inhibition of mitochondrial ribogenesis is also a promising anti-cancer approach [25, 26]. Mitoribogenesis can be hindered by drugs targeting POLRMT-mediated mt-rRNA transcription, like 2'C-Methyl-Adenosine (2’-C-MeA), as recently reported in acute myelogenous leukemia studies [27]. Moreover, due to the conservation between bacteria and mitochondria, antibiotics inhibiting bacterial protein synthesis (e.g. doxycycline) seem capable of inhibiting cancer cell proliferation by targeting mitochondrial translation [7, 10, 26].

In this study we provide evidence that MYC-induced rRNA transcription both in the nucleolus and mitochondrion concur to promote the proliferation of MYC-overexpressing cancer cells. Consistently, MYC overexpression sensitizes cells to the antiproliferative action of drugs targeting on one hand nucleolar Pol I-mediated rRNA transcription (e.g. CX-5461), and on the other hand, either POLRMT-mediated mitochondrial rRNA transcription (e.g. 2’-C-MeA) or mitoribosome function (e.g. doxycycline). Moreover, both 2’-C-MeA and doxycycline, when combined with CX-5461, induce a more effective antiproliferative action on MYC-overexpressing cells. Overall, targeting MYC-regulated rRNA transcription and ribogenesis in two distinct cell sites is an unconventional approach worth of consideration for treating MYC-driven cancer.

## RESULTS

### Evidence of MYC-induced proliferation consequent to upregulation of both “Pol I regulon” factors and rRNA transcription in human HFF fibroblasts

MYC is known to promote nucleolar rRNA transcription through direct activation of the Pol I transcription machinery [2–4] as well as activation of Pol II-mediated transcription of MYC-target genes encoding “Pol I regulon” factors, including RRN3, UBTF, TAF1C, and the Pol I subunits POLR1B and POLR1E [5, 6]. To start to assess the proliferation effects of MYC overexpression due to both Pol II-mediated transcription of “Pol I regulon” factors and Pol I-mediated rRNA transcription, we used a well-characterized model of MYC-overexpressing human fibroblasts (HFF-MYC) [3, 28, 29].

**Figure 1:**
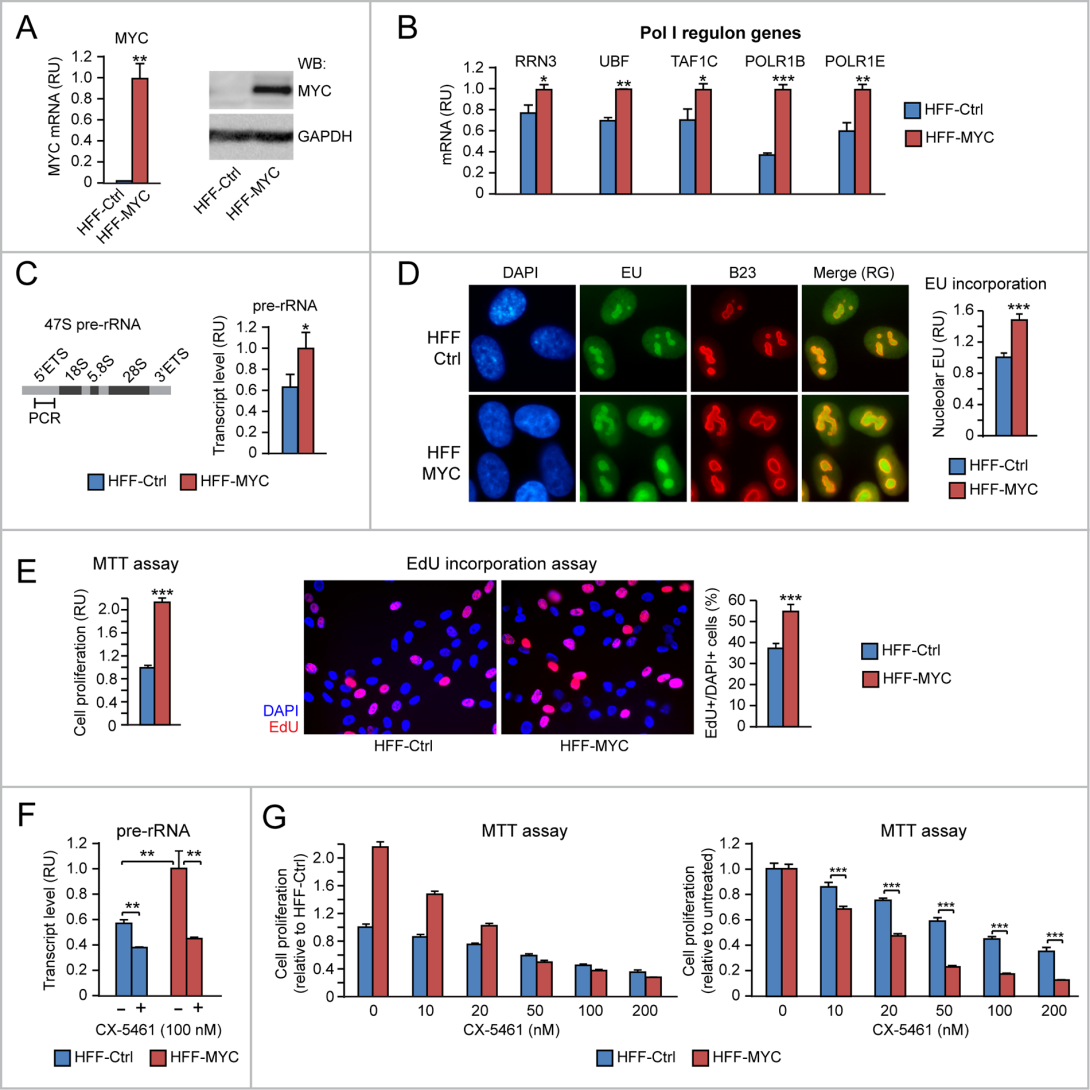
Evidence of MYC-induced proliferation consequent to upregulation of both “Pol I regulon” factors and rRNA transcription in human HFF fibroblasts **(A-B)** HFF-MYC fibroblasts overexpressing exogenous MYC (see qRT-PCR in A, left, and Western Blot in A, right) express significantly higher levels of “Pol I regulon” genes (assessed by q-RT-PCR) (B) relative to HFF-Ctrl fibroblasts. **(C-D)** HFF-MYC display increased nucleolar rRNA transcription relative to HFF-Ctrl, as measured by qRT-PCR of pre-rRNA 5’ETS (C) as well as by immunofluorescent quantification of EU incorporation in the B23-positive nucleolar compartment (D). Nucleolar EU incorporation was digitally quantified with Photoshop (see Materials and Methods for details). **(E)** MTT assay (left) and EdU incorporation analysis (middle and right) show that HFF-MYC cells proliferate significantly more than HFF-Ctrl cells. **(F-G)** Treatment with the Pol I inhibitor CX-5461, by reducing nucleolar rRNA transcription (F), inhibits proliferation of HFF-MYC cells significantly more than HFF-Ctrl cells (G). The chart in G, left, shows values normalized to untreated HFF-Ctrl; the chart in G, right, shows values normalized to each untreated cell line. ^*^p<0.05, ^**^p<0.01, ^***^p<0.001.

**Figure 2:**
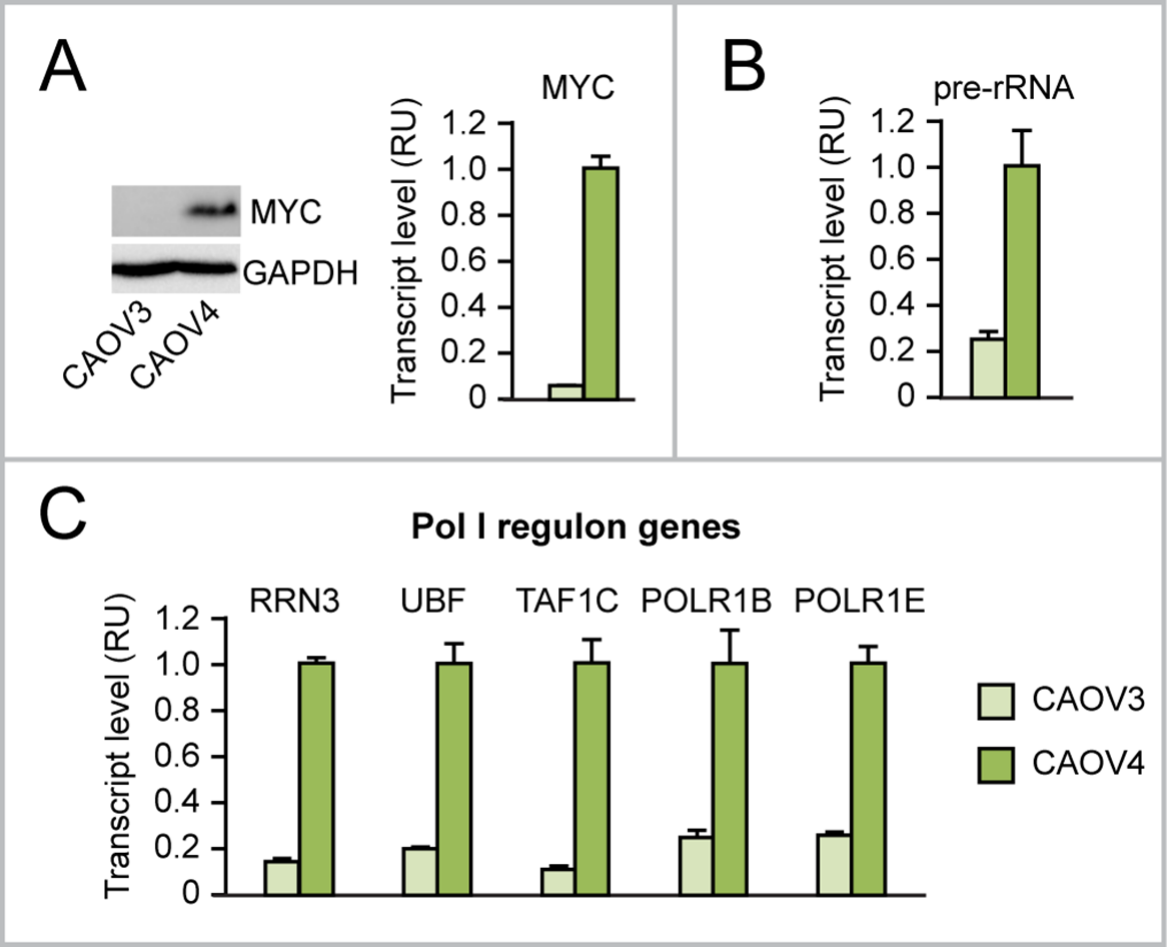
Evidence that MYC overexpression in ovarian cancer cells is associated with upregulation of rRNA and “Pol I regulon” factors **(A-C)** Endogenous MYC overexpression in CAOV4 ovarian cancer cells (see Western Blot in A, left, and qRT-PCR in A, right) is associated with increased rRNA transcription (assessed by pre-rRNA qRT-PCR) (B) and increased expression of “Pol I regulon” genes (assessed by qRT-PCR) (C) relative to CAOV3 ovarian cancer cells, with no MYC overexpression. All differences between CAOV4 and CAOV3 are significant (p<0.05).

HFF-MYC cells (Figure 1A) express higher levels of RRN3, UBF, TAF1C, POLR1B and POLR1E mRNAs relative to control HFF (HFF-Ctrl) (Figure 1B). Consistently, HFF-MYC also displayed upregulation of rRNA transcription, which was assessed by using two different strategies: 1) qRT-PCR of the 47S pre-rRNA 5'ETS, which is spliced out from mature rRNA (Figure 1C), and 2) quantitative analysis of 5-Ethynyl Uridine (EU) incorporation into newly synthesized RNA within the B23-positive nucleolar compartment (Figure 1D).

MYC overexpression significantly promoted HFF-MYC cell proliferation, which was evaluated both by both MTT assay (Figure 1E, left) and incorporation of 5-ethynyl-2′-deoxyuridine (EdU) into nascent DNA of cells in S phase (Figure 1E, middle and right). To further assess if upregulation of rRNA transcription contributes to MYC-induced cell proliferation, we treated cells with the Pol I inhibitor CX-5461, which prevents the formation of the Pol I pre-initiation complex [17]. Treatment with CX-5461 (100 nM) significantly counteracted MYC-induced rRNA transcription (assessed as pre-rRNA) in HFF-MYC cells (Figure 1F, red bars). CX-5461 also counteracted HFF-MYC proliferation in a dose-dependent manner (Figure 1G, left, red bars). To a lesser extent, CX-5461 inhibited rRNA transcription (Figure 1F, blue bars) and, consequently, proliferation (Figure 1G, left, blue bars) also in the HFF-Ctrl normal cell context. However, when HFF-MYC and HFF-Ctrl proliferation data were normalized to their respective untreated controls, HFF-MYC showed significantly higher sensitivity to the anti-proliferative action of CX-5461 relative to normal HFF-Ctrl (Figure 1G, right).

Overall, these preliminary results in a human fibroblast cell context indicate that MYC-induced proliferation can be traced to increased rRNA transcription due to: a) MYC-induced direct activation of Pol I, and b) MYC-induced upregulation of “Pol I regulon” factors. Moreover, MYC overexpression sensitizes HFF cells to the anti-proliferative action of the Pol I inhibitor CX-5461.

### Increased proliferation consequent to upregulation of both “Pol I regulon” genes and rRNA transcription in MYC-overexpressing human ovarian epithelial cells

According to The Cancer Genome Atlas, MYC amplification/upregulation is frequent in cancer, including ovarian cancer, where it is detected in over 30% of cases [30]. For this reason, we tested if MYC overexpression in ovarian cancer induces Pol I-mediated rRNA transcription as well as Pol II-mediated transcription of “Pol I regulon” genes. As shown here, CAOV4 ovarian cancer cells, which overexpress MYC (Figure 2A), display increased levels of rRNA transcription (assessed as pre-rRNA) (Figure 2B) and upregulation of “Pol I regulon” genes (Figure 2C) relative to CAOV3 ovarian cancer cells, which do not overexpress MYC.

**Figure 3:**
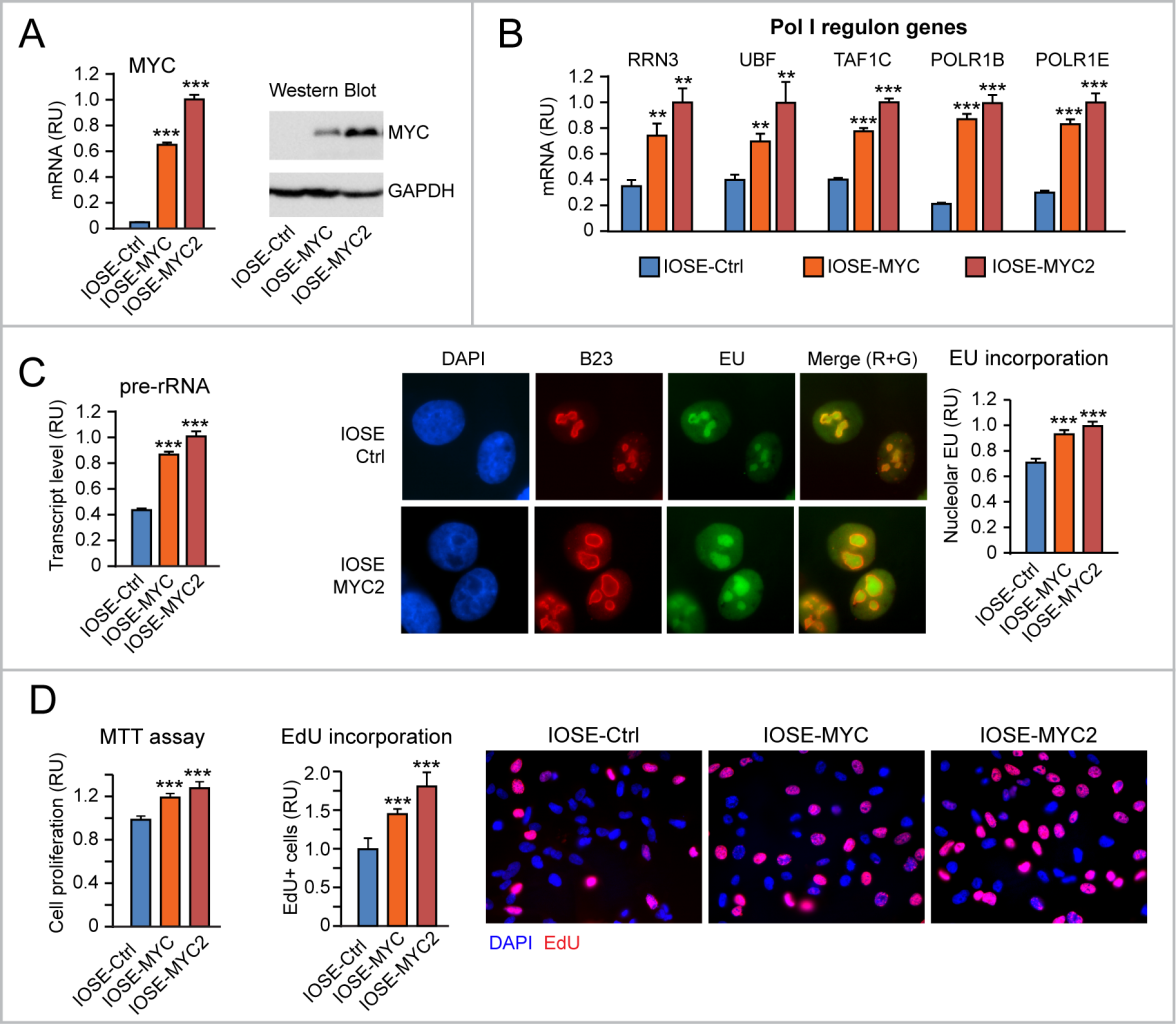
Increased proliferation consequent to upregulation of both “Pol I regulon” genes and rRNA transcription in MYC-overexpressing human ovarian epithelial cells **(A-B)** Increasing expression of exogenous MYC in IOSE ovarian epithelial cells (see qRT-PCR in A, left, and Western Blot in A, right) leads to a MYC dose-dependent upregulation of “Pol I regulon” genes (assessed by qRT-PCR) relative to control IOSE cells (B). **(C)** Exogenous MYC expression in IOSE cells leads to upregulation of rRNA transcription, assessed both by pre-rRNA qRT-PCR (left) and quantification of EU incorporation into the B23-positive nucleolar compartment (middle and left). Nucleolar EU incorporation was digitally quantified with Photoshop (see Materials and Methods for details). **(D)** Exogenous MYC expression promotes IOSE proliferation, as shown by both MTT assay (left) and EdU incorporation (middle and right). ^**^p<0.01, ^***^p<0.001.

Next, we set out to test the effects of different levels of MYC overexpression on nucleolar rRNA transcription in IOSE and HOSE human ovarian epithelial cells. Using telomerase-immortalized IOSE cells [31] we developed cells with increasing expression levels of exogenous MYC: IOSE-MYC, with MYC expression higher than control IOSE-Ctrl cells, and IOSE-MYC2, with a MYC overexpression level higher than IOSE-MYC (Figure 3A). The expression of “Pol I regulon” target genes (RRN3, UBF, TAF1C, POLR1B, POLR1E) was significantly higher in MYC-overexpressing IOSE lines than in IOSE-Ctrl, and correlated with MYC level (Figure 3B). Moreover, MYC-overexpressing IOSE displayed increased rRNA transcription, which was assessed both by pre-rRNA qRT-PCR (Figure 3C, left) and nucleolar EU incorporation (Figure 3C, middle and right). Consistently, we detected a MYC level-dependent increase of cell proliferation in IOSE-MYC and IOSE-MYC2 relative to IOSE-Ctrl (see MTT assay in Figure 3D, left, and EdU incorporation assay in Figure 3D, middle and right). We obtained similar findings also in SV40-immortalized HOSE cells (Supplementary Figure 1A-1D).

Thus, regardless of the cell context, increasing MYC overexpression seems sufficient to differentially foster cell proliferation by inducing Pol II-dependent transcription of “Pol I regulon” genes as well as Pol I-dependent rRNA synthesis.

### Additional evidence that upregulation of “Pol I regulon” genes contributes to promote cell proliferation in MYC-overexpressing IOSE cells

To mechanistically prove that increased proliferation of MYC-overexpressing IOSE cells was due, in part, to upregulation of “Pol I regulon” genes and rRNA transcription, we transiently transfected IOSE-MYC2 cells with short interfering RNAs (siRNAs) targeting either MYC, or either one of two key components of the “Pol I regulon”: RRN3 and UBF.

**Figure 4:**
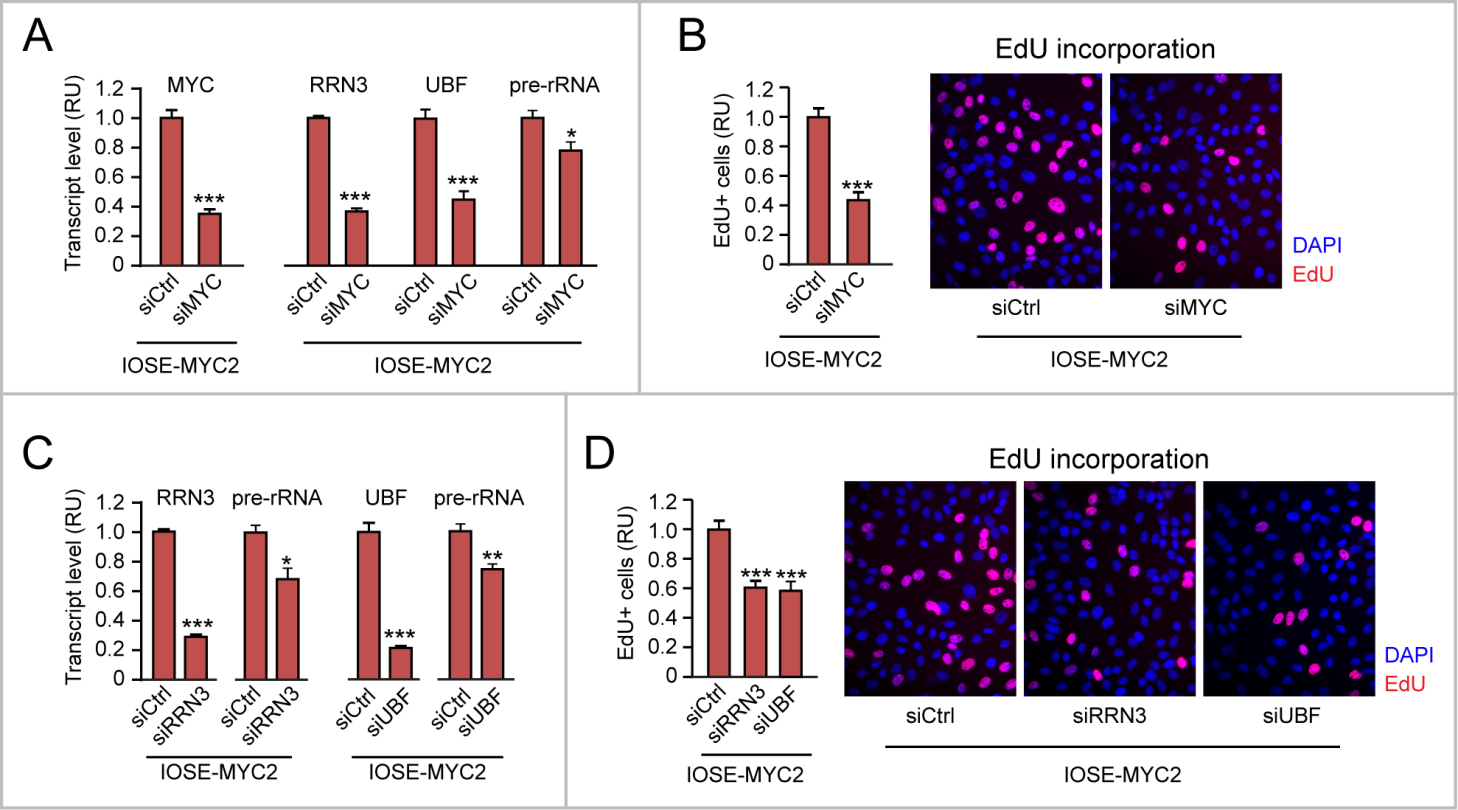
Additional evidence that upregulation of “Pol I regulon” genes contributes to promote cell proliferation in MYC-overexpressing IOSE cells **(A-B)** Decreasing the level of exogenous MYC in IOSE-MYC2 cells by transient siRNA (see qRT-PCR in A, left) is sufficient to significantly decrease the transcript levels of RRN3, UBF, and pre-rRNA (see qRT-PCR in A, right) as well as cell proliferation (see EdU incorporation in B). **(C-D)** Transient knock down of either RRN3 or UBF in IOSE-MYC2 significantly decreases pre-rRNA level (see qRT-PCR in C) and cell proliferation (see EdU incorporation in D). ^*^p<0.05, ^**^p<0.01, ^***^p<0.001.

As shown in Figure 4A, transient MYC knock down by siMYC in IOSE-MYC2 cells (left) resulted in downregulation of both RRN3 and UBF mRNAs, as well as in decreased pre-rRNA level (right) relative to cells transfected with control siRNA (siCtrl). Importantly, MYC knock down also counteracted MYC-induced proliferation of IOSE-MYC2 cells (see EdU incorporation assay in Figure 4B). In turn, transient knock down of either RRN3 (Figure 4C, left) or UBF (Figure 4C, right) in IOSE-MYC2 was sufficient to significantly reduce both rRNA transcription (see pre-rRNA qRT-PCR in Figure 4C, left and right), and cell proliferation (see EdU incorporation assay in Figure 4D).

Based on these findings, the pro-proliferative effects of MYC overexpression in human ovarian epithelial cells rely, at least in part, on MYC transcriptional ability to upregulate “Pol I regulon” genes.

### MYC overexpression sensitizes human ovarian epithelial cells to the anti-proliferative action of the Pol I inhibitor CX-5461

Selective Pol I inhibitors have been rapidly emerging as drugs capable of targeting increased rRNA synthesis [19, 32]. Based on preliminary evidence of an antiproliferative effect of the Pol I inhibitor CX-5461 in MYC-overexpressing HFF (see Figure 1G), we did test if MYC-induced rRNA transcription was also capable of sensitizing IOSE cells to the anti-proliferative action of CX-5461.

**Figure 5:**
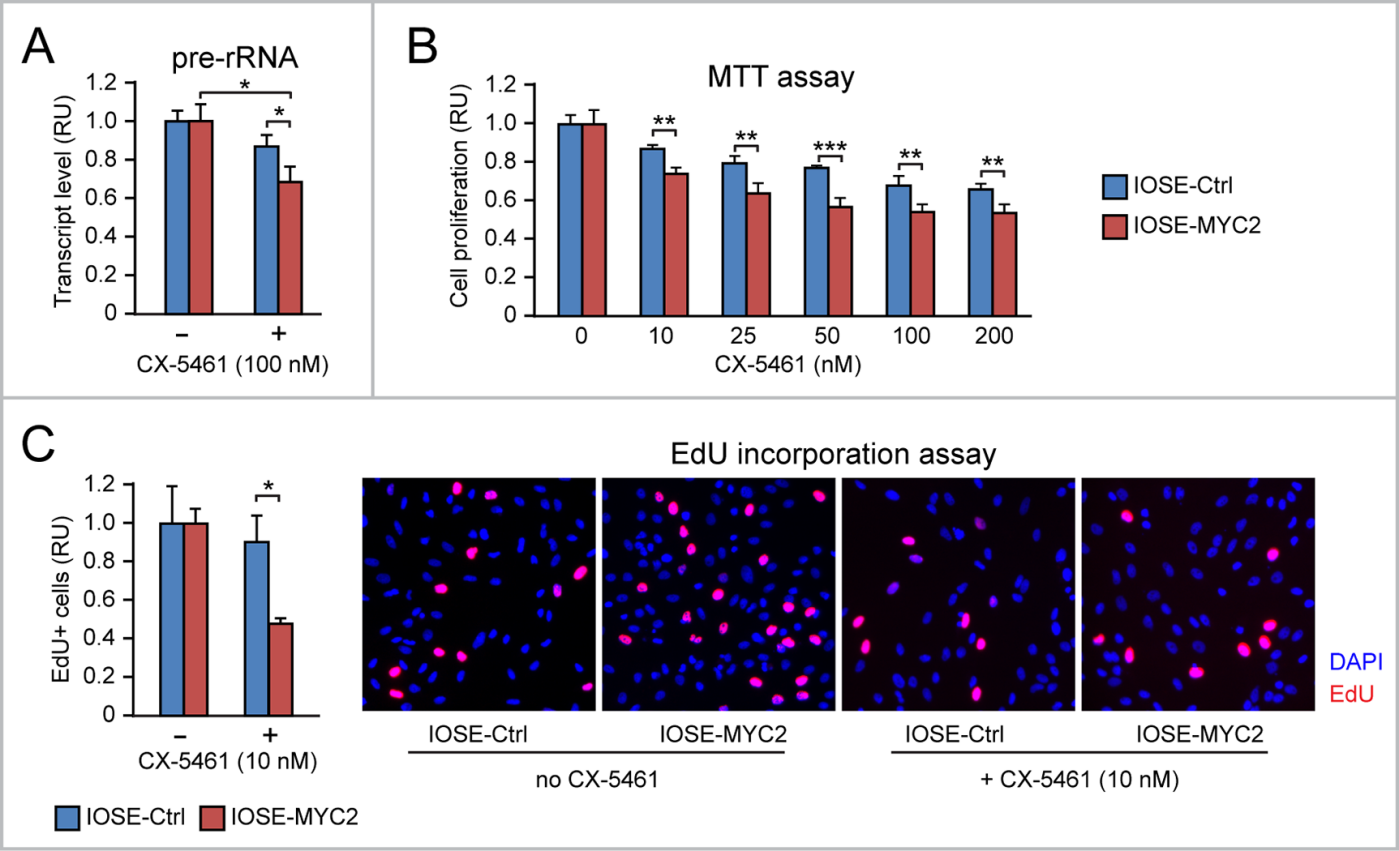
MYC overexpression sensitizes human ovarian epithelial cells to the anti-proliferative action of the Pol I inhibitor CX-5461 **(A)** The Pol I inhibitor CX-5461 reduces rRNA transcription (assessed by pre-rRNA qRT-PCR) significantly more in IOSE-MYC2 than in IOSE-Ctrl cells. **(B-C)** Consistently, IOSE-MYC2 are more sensitive than IOSE-Ctrl cells to the anti-proliferative action of CX-5461, as shown by both MTT assay (B) and analysis of EdU incorporation by immunofluorescence (C). ^*^p<0.05, ^**^p<0.01, ^***^p<0.001.

As shown in Figure 5A, CX-5461 (100 nM) reduced rRNA transcription (assessed by pre-rRNA quantitative RT-PCR) significantly more in IOSE-MYC2 relative to IOSE-Ctrl. Consistently, IOSE-MYC2 cells also displayed increased sensitivity to the anti-proliferative action of CX-5461 even at the lowest concentration (Figure 5B). Indeed by EdU incorporation we found that at 10 nM, CX-5461 reduced the number of cells in S phase significantly more in IOSE-MYC2 than in IOSE-Ctrl cells (Figure 5C). A similar antiproliferative response to CX-5461 was also found in HOSE cells (Supplementary Figure 1E).

Overall, these findings indicated that a) upregulation of Pol I-mediated rRNA transcription contributes to MYC-induced proliferation in ovarian epithelial cells, and b) MYC-overexpressing human ovarian epithelial cells are more sensitive to the anti-proliferative action of the Pol I inhibitor CX-5461.

### Upregulation of genes involved in both mitochondrial rRNA transcription and mitoribogenesis contributes to MYC-induced HFF proliferation

Recent studies in different cell contexts indicate that MYC can promote mitochondrial ribogenesis by activating Pol II-dependent transcription of the mitochondrial RNA polymerase POLRMT and the mitochondrial transcription factor TFAM [7, 8]. Further, MYC can induce Pol II-dependent transcription of several mitoribosome proteins (e.g. PTCD3, MRPS5, and MRPS27) [10].

**Figure 6:**
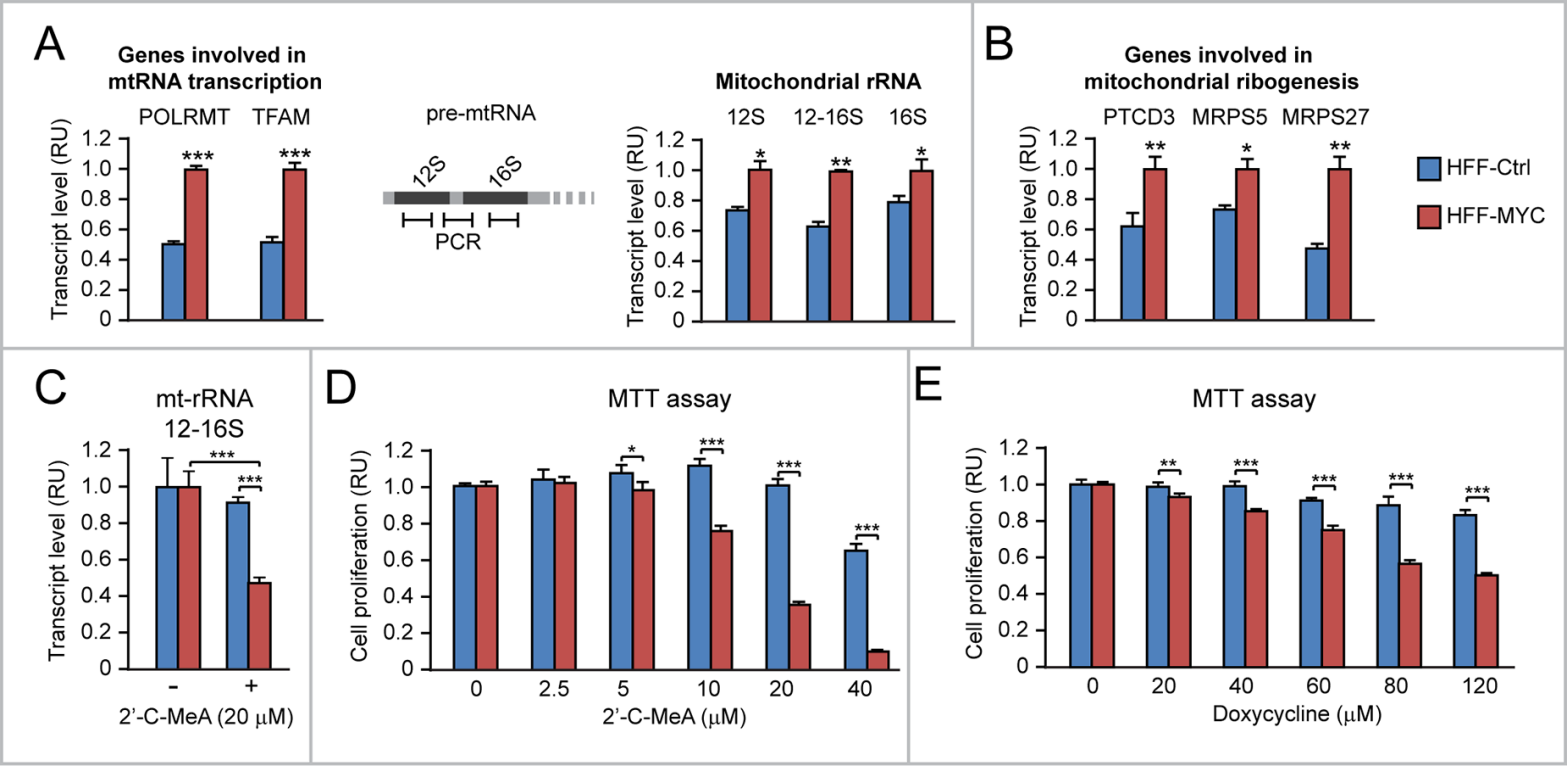
Upregulation of mitochondrial rRNA transcription and mitoribosome proteins contributes to MYC-induced HFF proliferation **(A)** HFF-MYC, overexpressing exogenous MYC, display upregulation of genes involved in mitochondrial RNA transcription (left), as well as increased mitochondrial rRNA levels (right), which were quantified by qRT-PCR of the 12S, 16S, or 12-16S regions of pre-mtRNA (see scheme in the middle). **(B)** HFF-MYC cells also display upregulation of genes encoding the mitochondrial ribosomal proteins PTCD3, MRPS5, and MRPS27. **(C-D)** The POLRMT inhibitor 2’-C-MeA inhibits mitochondrial rRNA transcription (assessed by qRT-PCR of the 12-16S mt-rRNA) (C) and cell proliferation (assessed by MTT assay) (D) significantly more in HFF-MYC relative to HFF-Ctrl. **(E)** HFF-MYC are more sensitive than HFF-Ctrl to the antiproliferative action of doxycycline, which inhibits mitoribosome function. ^*^p<0.05, ^**^p<0.01, ^***^p<0.001.

We used the HFF model to assess whether the effects of MYC overexpression on proliferation can be traced to either increased mt-rRNA transcription or upregulation of mitoribosome proteins. Relative to HFF-Ctrl, HFF-MYC cells displayed increased levels of both POLRMT and TFAM mRNAs (Figure 6A, left), as well as increased levels of three pre-mtRNA regions (Figure 6A, middle and right): i) a region within the 12S mitochondrial RNA, ii) a region within the 16S mtRNA, and iii) a region across the 12S and 16S mt-rRNA. Since the 12S and 16S RNAs are subsequently spliced out to form mature mitochondrial rRNAs, this last region is present only in mt-rRNA precursors, thus indirectly reflecting the rate of mt-rRNA transcription. In addition, HFF-MYC also displayed increased transcript levels of PTCD3, MRPS5, and MRPS27 mitoribosome proteins relative to HFF-Ctrl (Figure 6B), thus confirming that, also in this cell context, MYC overexpression can promote mitoribosome formation by concomitantly enhancing both mt-rRNA transcription and expression of mitochondrial ribosomal proteins.

To test the contribution of MYC-induced mt-rRNA transcription and mitoribogenesis to MYC-induced proliferation, first we treated both HFF-Ctrl and HFF-MYC cells with 2'-C-methyladenosine (2’-C-MeA), which inhibits POLRMT-mediated transcription of mt-rRNA [33]. As shown in Figure 6C, treatment with 2’-C-MeA (20 μM) significantly inhibited mt-rRNA transcription (assessed by qRT-PCR of the 12-16S region of the pre-mtRNA) in HFF-MYC. Consistently, 2’-C-MeA also inhibited HFF-MYC proliferation in a dose-dependent fashion (see in Figure 6D, red bars). Moreover, when compared to HFF-Ctrl, HFF-MYC clearly displayed increased sensitivity to the anti-proliferative action of 2’-C-MeA (Figure 6D).

Second, we treated both HFF-Ctrl and HFF-MYC cells with doxycycline, a tetracycline that targets the 28S subunit of mitochondrial ribosomes, which is homologous to the 30S subunit of bacterial ribosomes. Doxycycline inhibited HFF-MYC proliferation in a dose-dependent fashion, and its anti-proliferative effects were significantly greater in the HFF-MYC cell context than in the HFF-Ctrl cell context (Figure 6E).

These preliminary findings in the HFF model showed that: i) MYC-induced mt-rRNA transcription and mitoribogenesis contribute to promote HFF-MYC proliferation; ii) MYC overexpression sensitizes HFF-MYC cells to the anti-proliferative action of drugs inhibiting either mt-rRNA transcription (2’-C-MeA) or mitoribosome function (doxycycline).

### MYC-induced upregulation of either mt-rRNA transcription or mitoribogenesis in human ovarian epithelial cell contexts contributes to promote proliferation

**Figure 7:**
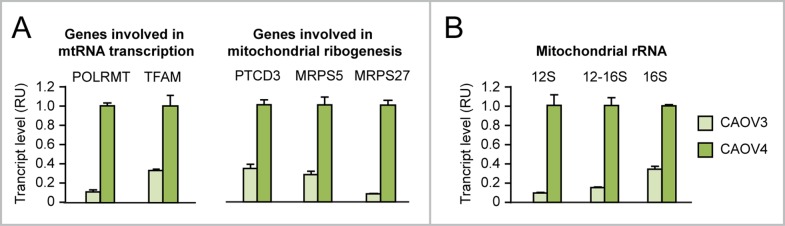
Evidence that MYC overexpression in ovarian cancer cells is associated with upregulation of genes involved in mitochondrial rRNA transcription and ribogenesis **(A-B)** qRT-PCR showing that CAOV4 ovarian cancer cells, which overexpress endogenous MYC, display upregulation of genes involved in mitochondrial rRNA transcription (A, left) and ribogenesis (A, right) as well as increased levels of 12S, 16S, and 12-16S mitochondrial rRNA (B) relative to CAOV3 cells, which do not overexpress MYC. All differences between CAOV4 and CAOV3 are significant (p<0.05).

We found evidence that, relative to CAOV3 ovarian cancer cells, which do not overexpress MYC, CAOV4 ovarian cancer cells, which overexpress MYC (see Figure 2A), display a) upregulation of both MYC-target genes involved in mt-rRNA transcription (POLRMT and TFAM) and MYC-target genes encoding mitoribosome proteins (PTCD3, MRPS5, MRPS27) (Figure 7A) as well as b) increased mitochondrial rRNA synthesis (Figure 7B). For this reason, we set out to test whether MYC ectopic overexpression in human ovarian epithelial cell contexts (IOSE and HOSE) leads to increased mt-rRNA synthesis and affects mitoribogenesis.

**Figure 8:**
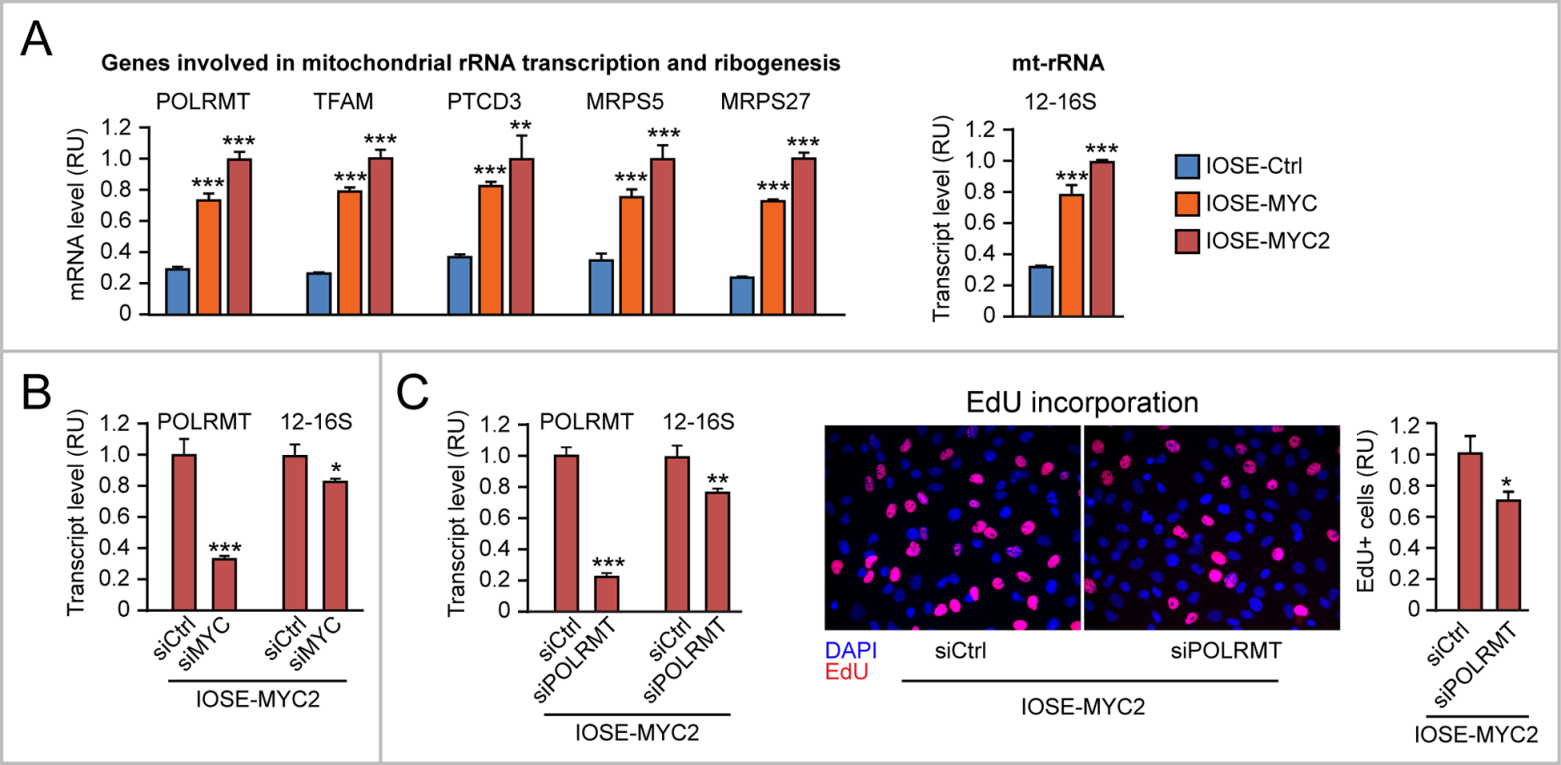
MYC-induced upregulation of mitochondrial rRNA transcription and ribogenesis contributes to promote proliferation of human ovarian epithelial cells **(A)** qRT-PCR showing MYC dose-dependent upregulation of genes involved in mitochondrial rRNA transcription and ribogenesis (left) as well as 12-16S mt-rRNA (right) in IOSE-MYC and IOSE-MYC2 cells relative to IOSE-Ctrl. **(B)** Transient MYC knock down by siRNA significantly reduces both POLRMT and mt-rRNA transcription (assessed by 12-16S mt-rRNA qRT-PCR) in IOSE-MYC2. **(C)** Transient POLRMT knock down in IOSE-MYC2 cells, by reducing mt-rRNA transcription (left), leads to decreased proliferation (assessed by EdU incorporation) (middle and right). ^*^p<0.05, ^**^p<0.01, ^***^p<0.001.

Using the panel of IOSE cells, we detected MYC level-dependent upregulation of POLRMT, TFAM, PTCD3, MRPS5, and MRPS27 mRNAs (Figure 8A, left), as well as increased 12-16S mt-rRNA level (Figure 8A, right) in IOSE-MYC and IOSE-MYC2 cells relative to IOSE-Ctrl cells. In addition, transient MYC knock down in IOSE-MYC2 significantly reduced both POLRMT and 12-16S mt-rRNA transcript levels (Figure 8B). In turn, POLRMT knock down in IOSE-MYC2 decreased both mt-rRNA transcription (Figure 8C, left) and cell proliferation (assessed by EdU incorporation, Figure 8C, middle and right). Similar findings were obtained in the HOSE cell context (Supplementary Figure 2A-2B).

Apparently, MYC ectopic overexpression in different human ovarian epithelial cell contexts promotes mt-rRNA transcription and mitoribogenesis by upregulating MYC-target genes that are involved in these two processes. Moreover, MYC-induced mt-rRNA transcription and mitoribogenesis contribute to MYC-induced cell proliferation.

### Antiproliferative effect of 2’-C-MeA and doxycycline, alone or in combination with CX-5461, on MYC overexpressing ovarian epithelial cells

Recently, it was reported that selective inhibition of either mt-rRNA transcription or mitoribosome function exerts an antiproliferative effect on MYC-overexpressing cancer cells [7, 10].

**Figure 9:**
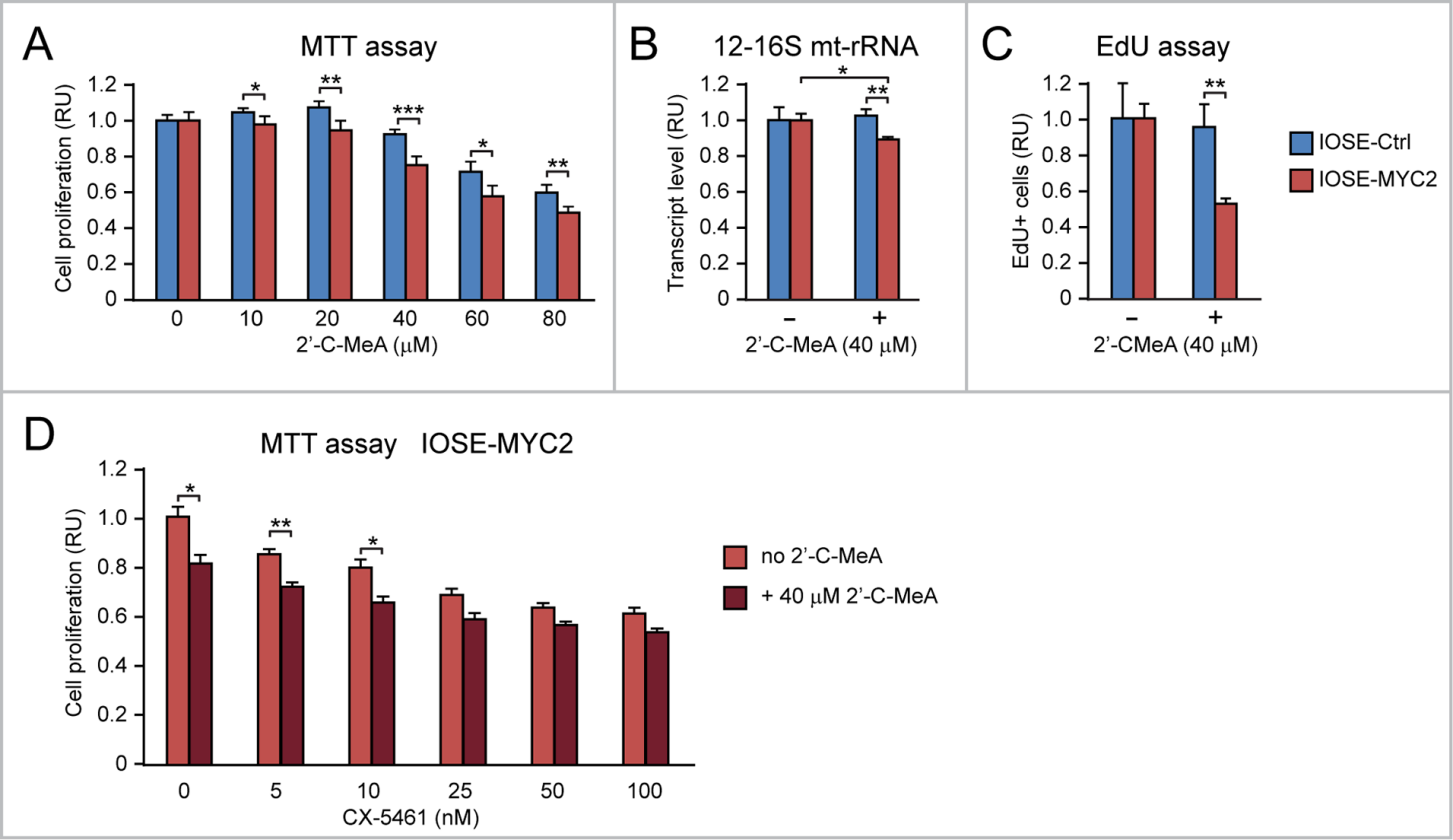
MYC overexpression sensitizes human ovarian epithelial cells to the anti-proliferative action of 2’-C-MeA, alone or in combination with CX-5461 **(A)** 2’-C-MeA inhibits cell proliferation (assessed by MTT assay) significantly more in IOSE-MYC2 that IOSE-Ctrl. **(B-C)** 2’-C-MeA (40 μM) reduces both mt-rRNA transcription (assessed by qRT-PCR of 12-16S mt-rRNA) (B) and the number of cells in S phase (assessed by EdU incorporation) (C) significantly more in IOSE-MYC2 than in IOSE-Ctrl cells. **(D)** MTT assay showing that 2’-C-MeA (40 μM) significantly enhances the antiproliferative effect of CX-5461 (5-10 nM) in IOSE-MYC2 cells. ^*^p<0.05, ^**^p<0.01, ^***^p<0.001.

First, we found by MTT assay that IOSE-MYC2, relative to IOSE-Ctrl cells (Figure 9A), display increased sensitivity to the antiproliferative action of the POLRMT inhibitor 2’-C-MeA. Similar findings were also obtained in HOSE-MYC relative to HOSE-Ctrl (Supplementary Figure 2C). The 2’-C-MeA concentration of 40 μM, which determined the most significant difference between IOSE-MYC2 and IOSE-Ctrl proliferation in the MTT assay, also decreased 12-16S mt-rRNA transcription (Figure 9B) and the number of cells in S phase (assessed by EdU incorporation) (Figure 9C) significantly more in IOSE-MYC2 versus IOSE-Ctrl proliferation. Moreover, when we tested the effect of 40 μM 2’-C-MeA in combination with CX5461 (ranging from 5 nM to 100 nM) in IOSE-MYC2 cells, we found that 2’-C-MeA significantly enhanced the antiproliferative effect of CX-5461 low concentrations (5-10 nM) (Figure 9D).

**Figure 10:**
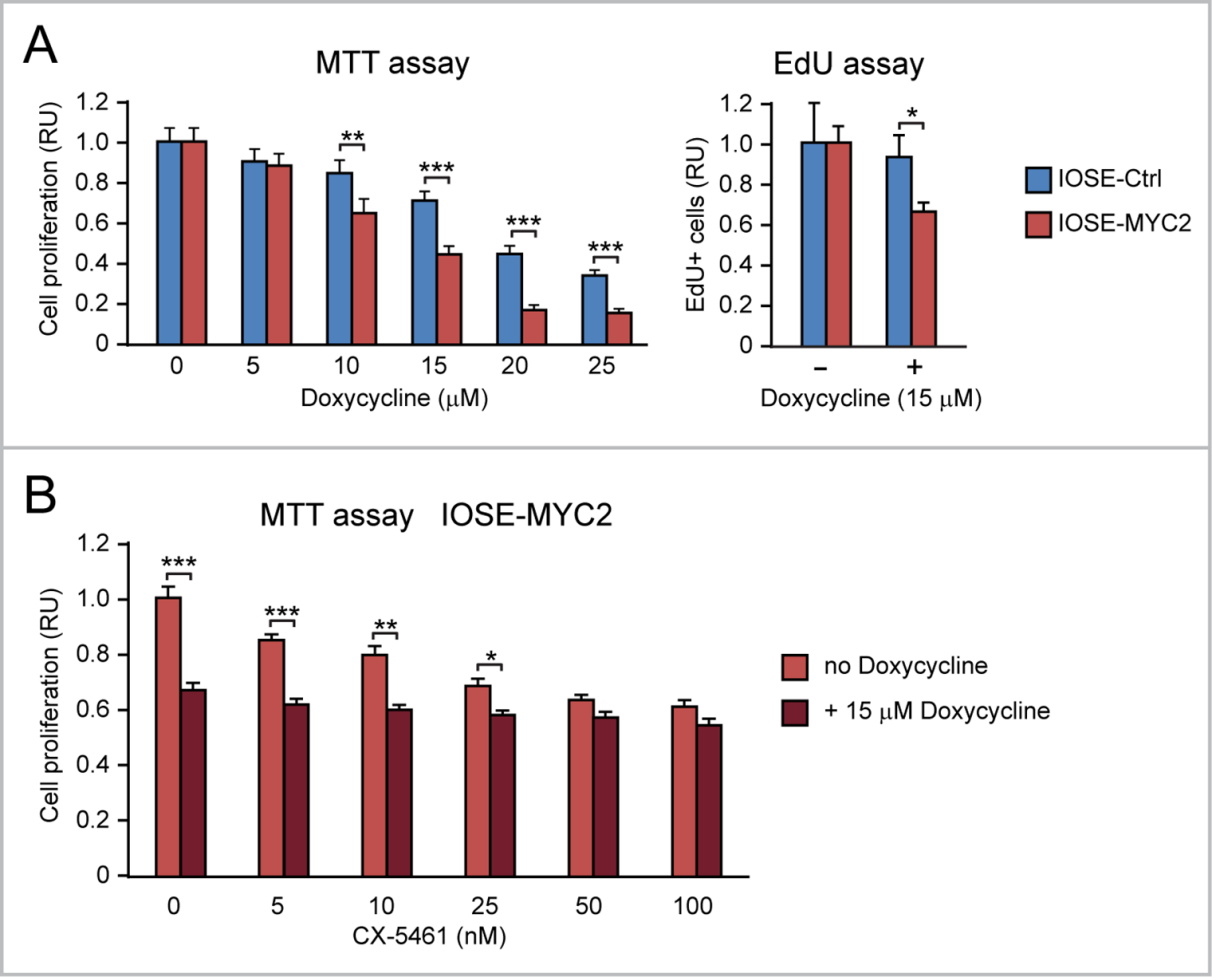
MYC overexpression sensitizes human ovarian epithelial cells to the anti-proliferative action of doxycycline, alone or in combination with CX-5461 **(A)** Doxycycline inhibits cell proliferation (assessed both by MTT assay, shown on the left, and EdU incorporation assay, shown on the right) significantly more in IOSE-MYC2 than in IOSE-Ctrl. **(B)** MTT assay showing that doxycycline (15 μM) significantly enhances the antiproliferative effect of CX-5461 (5-25 nM) in IOSE-MYC2 cells. ^*^p<0.05, ^**^p<0.01, ^***^p<0.001.

Second, by MTT assay we found that IOSE-MYC2 versus IOSE-Ctrl (Figure 10A, left), as well as HOSE-MYC versus HOSE-Ctrl (Supplementary Figure 2D), displayed increased sensitivity to the anti-proliferative action of doxycycline across a range of concentrations (0-25 μM). The doxycycline concentration of 15 μM showed the most significant difference between IOSE-MYC2 and IOSE-Ctrl proliferation both in the MTT assay (Figure 10A, left) and in the EdU incorporation assay (Figure 10A, right). Remarkably, when we tested the effect of 15 μM doxycycline in combination with CX5461 (ranging from 5 nM to 100 nM) on IOSE-MYC2 cells, we found that doxycycline significantly enhanced the antiproliferative effect of CX-5461 at concentrations between 5 and 25 nM (Figure 10B).

Apparently, MYC-overexpression sensitized ovarian epithelial cells to both 2’-C-MeA and doxycycline, which target, respectively, mt-rRNA transcription and mitoribosome function. Moreover both 2’-C-MeA and doxycycline potentiated the antiproliferative effect of low doses of the CX-5461 inhibitor of Pol I-mediated nucleolar rRNA transcription.

### Both 2’-C-MeA and doxycycline can enhance the antiproliferative effects of CX-5461 in MYC-overexpressing ovarian cancer cells

**Figure 11:**
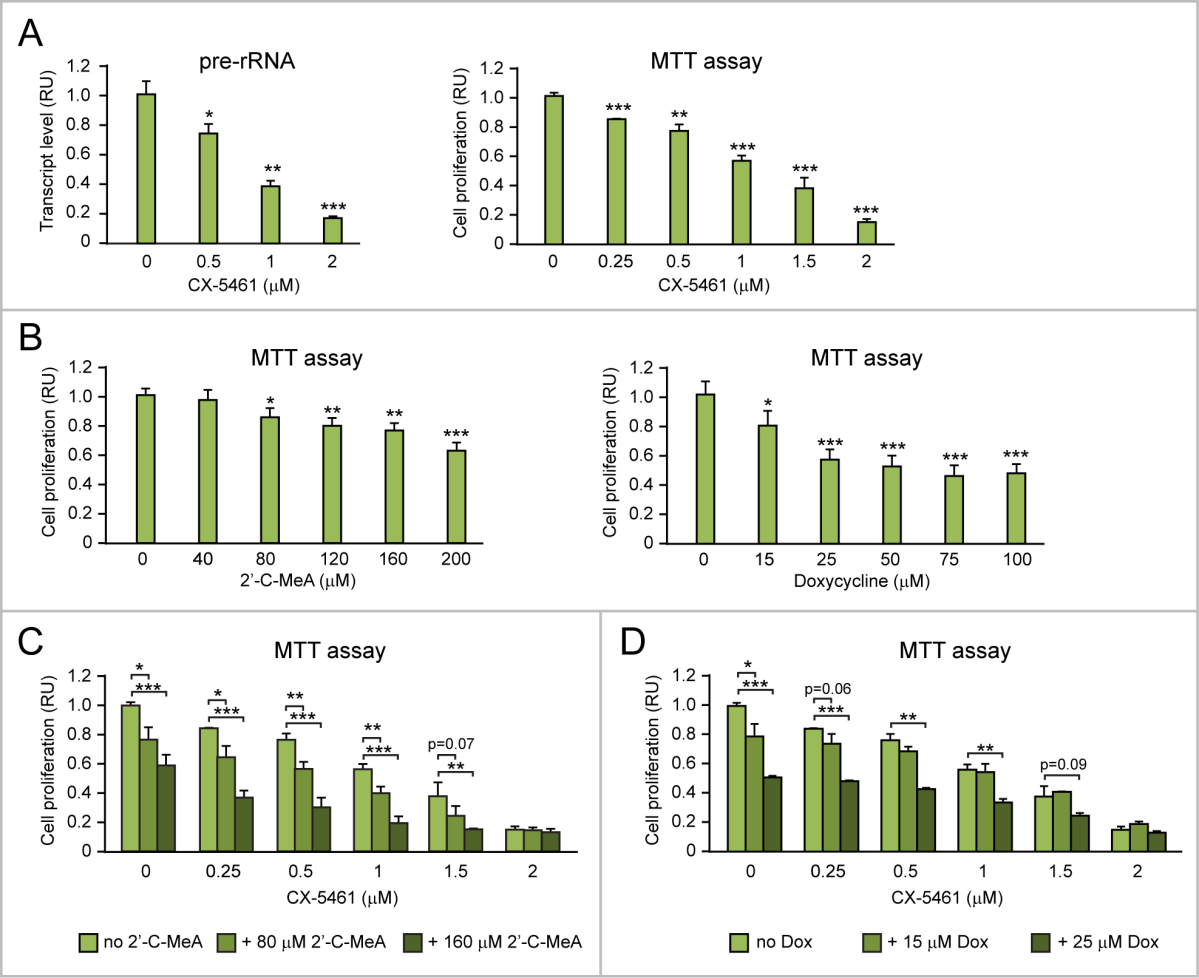
Both 2’-C-MeA and doxycycline can enhance the antiproliferative effects of CX-5461 in MYC-overexpressing ovarian cancer cells **(A)** CX-5461, by inhibiting Pol I-mediated rRNA transcription (assessed by pre-rRNA qRT-PCR, left), significantly hinders the proliferation of MYC-overexpressing CAOV4 ovarian cancer cells (assessed by MTT assay, right). **(B)** Both inhibition of mt-rRNA transcription by 2’C-MeA (left) and inhibition of mitoribosome function by doxycycline (right) significantly decrease CAOV4 cell proliferation. **(C-D)** Both 2’-C-MeA (C) and doxycycline (D) enhance the antiproliferative effects of CX-5461 in CAOV4 cells. ^*^p<0.05, ^**^p<0.01, ^***^p<0.001.

In this study we found that CAOV4 ovarian cancer cells overexpressing endogenous MYC show upregulation of nucleolar rRNA and “Pol I regulon” genes (Figure 2) as well as increased mitochondrial rRNA transcription and upregulation of MYC-target genes involved in mitochondrial rRNA transcription and ribogenesis (Figure 7). Dose-dependent inhibition of Pol I-mediated rRNA transcription with CX-5461 (Figure 11A, left) significantly reduced CAOV4 cell proliferation (Figure 11A, right). Treatment with either 2’C-MeA (Figure 11B, left) or doxycycline (Figure 11B, right) also led to dose-dependent inhibition of CAOV4 cell proliferation. Moreover, the anti-proliferative effects of CX-5461 on CAOV4 was significantly enhanced by co-treatment with either 2’C-MeA (80 or 160 μM, Figure 11C) or doxycycline (15 or 25 μM, Figure 11D), which were among the lowest concentrations able to induce significant inhibition of CAOV4 proliferation. In conclusion, combining CX-5461 with drugs inhibiting either mt-rRNA transcription (e.g. 2’C-MeA) or mitoribosome function (e.g. doxycycline) seems to be an effective antiproliferative strategy to treat MYC-overexpressing ovarian cancer.

## DISCUSSION

**Figure 12:**
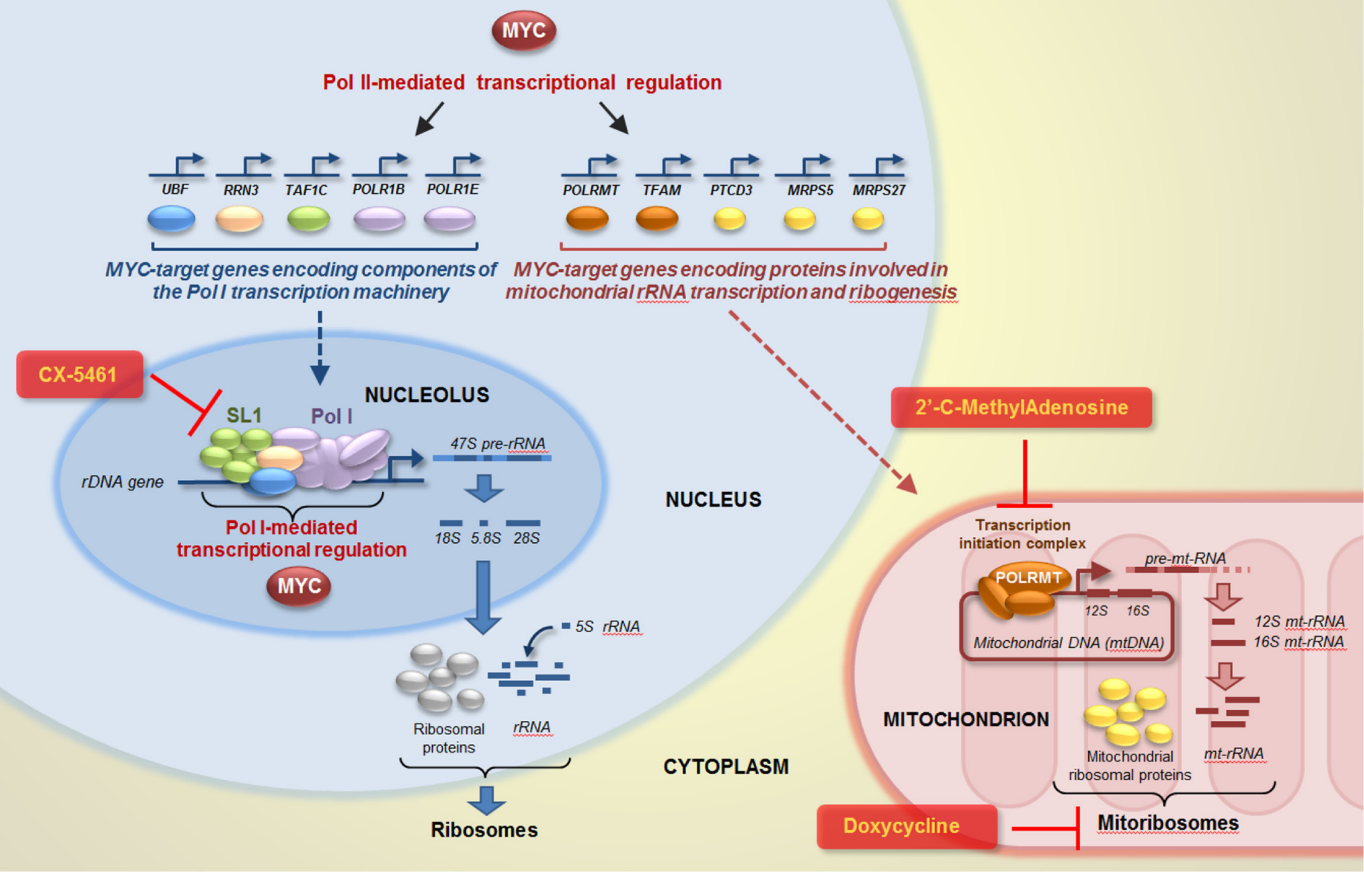
Targeting nucleolar and mitochondrial rRNA transcription and ribogenesis to curb MYC-induced cancer cell proliferation MYC can promote nucleolar rRNA transcription by directly activating Pol I-mediated transcription of ribosomal genes, as well as by upregulating Pol II-mediated transcription of “Pol I regulon” genes. Concomitantly, MYC can also promote mitoribosome synthesis by upregulating Pol II-mediated transcription of genes encoding factors necessary for mitochondrial rRNA transcription (POLRMT and TFAM) and mitochondrial ribosomal proteins (PTCD3, MRPS5, and MRPS27). Drugs inhibiting Pol I (e.g. CX-5461), mitochondrial rRNA transcription (e.g. 2’-C-MeA), or mitoribosome function (e.g. doxycycline) could be used, alone or in combination, to target MYC-overexpressing cancer cells, such as ovarian cancer cells.

Increasing knowledge of MYC-coordinated regulation of functions involved in both nucleolar and mitochondrial rRNA transcription and ribogenesis can open new avenues to treat MYC-driven cancer. In this study, we provide evidence of the added value of pharmacological targeting mitochondrial POLRMT-mediated mt-rRNA transcription or mitoribosome translational function in conjunction with a well-established inhibitor of nucleolar Pol I-mediated rRNA transcription in MYC-overexpressing (cancer) cells (Figure 12).

Evidence that nucleolar rRNA transcription and ribogenesis are frequently upregulated in cancer due to different etiological factors has led to the development of novel small molecules capable of inhibiting Pol I [15, 19, 34, 35]. In particular, one of these molecules, CX-5461, which targets Pol I-mediated rRNA transcription, proved to be effective in *in vitro* and preclinical MYC-driven cancer cell models [16, 21, 22]. CX-5461 is currently tested in clinical trials for advanced hematological malignancies (Australian New Zealand Clinical Trials Registry 12613001061729) and solid tumors (ClinicalTrials.gov Identifier NCT02719977), and its use could be extended to clinical trials of MYC-driven solid tumors, including breast, ovarian and prostate cancer [19, 24, 32].

Interestingly, CX-5461 was shown to be even more effective when used in combination with other drugs, such as drugs targeting PI3K-AKT-mTORC1-dependent nucleolar ribosome biogenesis and translation [21]. By the same tenet, we reasoned that CX-5461 could also be more effective if combined with drugs targeting mitochondrial ribogenesis processes and translation in MYC-overexpressing cells. Indeed, recent studies highlighted the anticancer potential of several antiviral and antibacterial molecules capable of targeting either POLRMT-mediated mitochondrial rRNA transcription or mitoribosome function [7, 10, 26]. Specifically, there are several ribonucleosides analogues, originally developed for antiviral therapy, which are substrates and inhibitors of human mitochondrial RNA polymerase (POLRMT) [33], and can be repurposed for cancer therapy. In preclinical studies the 2-C-methyladenosine ribonucleoside (2’-C-MeA) was shown to reduce mitochondrial gene expression and increase cell death in acute myelogenous leukemia (AML), while treatment of normal human hematopoietic cells did not alter clonogenic growth [27]. Due to the ancestral relationship between bacteria and mitochondria, several classes of antibiotics, including tetracyclines (e.g. Doxycycline and Tigecycline) capable of targeting mitoribosome function, are also re-evaluated as potential anti-cancer drugs ([26] and references within).

This study provides the proof of concept that available drugs targeting mitochondrial ribogenesis or mitoribosome function can improve the anticancer effects of emerging new drugs capable of selectively targeting nucleolar Pol I rRNA synthesis in cancer and, in particular, MYC-driven cancer of different histotypes due to concomitant MYC deregulation of both nucleolar and mitochondrial rRNA synthesis and ribogenesis processes.

Based on preliminary observations that ovarian cancer cells overexpressing MYC (CAOV4) displayed upregulation of POLRMT and mt-rRNA synthesis relative to ovarian cancer cells that do not overexpress MYC (CAOV3), we assessed the antiproliferative effects of targeting POLRMT-mediated mt-rRNA transcription in two human ovarian epithelial cell contexts (IOSE and HOSE), and in human fibroblasts (HFF) overexpressing MYC. Consistent with genetic studies (either MYC or POLRMT knock down), we found that the POLRMT inhibitor 2’-C-MeA significantly reduced both mt-rRNA transcription and the number of cells in S phase. Remarkably, even if MYC-overexpressing ovarian epithelial cells were more sensitive to the anti-proliferative action of the Pol I inhibitor CX-5461 alone, 2’-C-MeA significantly enhanced the effects of CX-5461. Indeed, in the presence of 2’-C-MeA, CX-5461 exerted an anti-proliferative effect at lower concentrations (5-10 nM).

Further, based on the fact that MYC upregulation induces PTCD3, MRPS5, and MRPS27 proteins necessary for mitoribogenesis in MYC-overexpressing CAOV4 cancer cells, as well as in HFF fibroblast and different ovarian epithelial cell contexts with MYC overexpression, we tested if doxycycline, a tetracycline that targets the 28S subunit of mitochondrial ribosomes, was capable of reducing MYC-induced proliferation. Indeed, we found that MYC-overexpressing fibroblasts and ovarian epithelial cells were more sensitive than control cells to the anti-proliferative action of doxycycline alone. Moreover, doxycycline, when used in combination with low doses of CX-5461 Pol I inhibitor, enhanced CX-5461 antiproliferative effect in MYC overexpressing ovarian epithelial cells. In the same cell contexts, we also tested Tigecycline, another tetracycline that was recently shown to curb proliferation of MYC-overexpressing lymphoma and osteosarcoma cells [7, 10], but we did not achieve the anti-proliferative effects of doxycycline (data not shown).

Finally, we found that CX-5461 significantly inhibits CAOV4 ovarian cancer cell proliferation, and that the anti-proliferative effect of CX-5461 is significantly enhanced by co-treatment with either 2’C-MeA or doxycycline. Since CX-5461 was recently shown to have a positive effect on both chemoresistant ovarian epithelial cells and patient derived xenografts [36], it is possible that drugs targeting mt-rRNA and mitoribogenesis, when combined with drugs inhibiting Pol I like CX-5461, can be also effective in chemoresistant MYC-overexpressing cancer cell populations.

Overall these findings indicate that leveraging a) MYC-regulated rRNA transcription by RNA polymerases in distinct cell sites (nucleolar Pol I and mitochondrial POLRMT) and b) other MYC-regulated processes and functions of ribogenesis and mitoribogenesis, can be an offbeat approach to control MYC-driven cancers.

## MATERIALS AND METHODS

### Cells and cell culture

HFF-Ctrl (originally named HFF-pB) and HFF-MYC human foreskin fibroblast cell lines, kindly obtained from Dr. C. Grandori, Fred Hutchinson Cancer Research Center, Seattle, and previously described in [3, 28], were grown in DMEM plus 10% FBS (Thermo Fisher). CAOV3 and CAOV4 ovarian cancer cells (both from ATCC, Manassas, VA) were grown in DMEM plus 10% FBS and MCDB105/Media199 1:1 plus 15% FBS, respectively. Telomerase-immortalized human ovarian surface epithelial cells (IOSE, clone C21), kindly provided by Dr. F. Balkwill, Barts Cancer Institute, Queen Mary University of London, London, were grown in MCDB105/Media199 1:1 plus 15% FBS supplemented with 5 μg/ml insulin (Sigma), 10 ng/ml human EGF (Peprotech), 0.5 μg/ml hydrocortisone (Sigma), and 34 μg protein/ml BPE (Lonza) [31]. T1074 SV40-immortalized human ovarian epithelial cells (in this study referred to as HOSE) were purchased from ABM (Richmond, BC, Canada) and grown in MCDB105/Media199 1:1 plus 15% FBS. All cell lines were grown in a humidified incubator with 5% CO_2_ at 37°C.

IOSE cells stably overexpressing different levels of ectopic MYC (IOSE-MYC and IOSE-MYC2) were developed by lentiviral infection. First, human MYC cDNA was subcloned from pCDH-puro-MYC (Addgene) into the BamH I/Xba I restriction sites of pCDH-Hygro (CD515B-1) (System Biosciences). Second, pCDH-Hygro-MYC was co-transfected along with VSV-G and DeltaR plasmids (both provided by Dr. K. Gurova, Roswell Park Cancer Institute, Buffalo, NY) into HEK293 cells by using lipofectamine LTR (Thermo Fisher) in order to produce lentiviral particles. Medium containing lentiviral particles was harvested after 48h and 72h and filtered through a 0.45 μ filter. Lentiviral titer was assessed by using Lenti-X Go Stix (Clontech). Finally, 40-50% confluent IOSE cells were infected with medium containing lentiviral particles diluted 1:2 (IOSE-MYC2) or 1:3 (IOSE-MYC) with growth medium, in the presence of polybrene at a final concentration of 8 μg/ml. The infection was repeated after 24h. After two rounds of infection, cells were trypsinized, seeded in flasks and selected with 0.2 mg/ml hygromycin. In parallel, IOSE were infected with empty pCDH-Hygro to obtain IOSE-Ctrl cells. HOSE stably overexpressing ectopic MYC (HOSE-MYC2) were developed by lentiviral infection as described above using pCDH-puro-MYC for lentiviral production and using puromycin (0.5 μg/ml) for selection. Control HOSE were developed by infection with empty pCDH-puro. HFF-pB and HFF-MYC were authenticated by PCR detection of pBABE and pBABE-MYC, respectively. IOSE and HOSE were fingerprinted by STR analysis.

### Drugs and treatments

CX-5461 (ApexBio) was dissolved in 50 mM NaH_2_PO_4_ (pH 4.5) to obtain a 1 mM stock solution. 2’-C-Methyladenosine (Santa Cruz Biotechnologies) was dissolved in DMSO to obtain a 100 mM stock solution. Doxycycline (Fisher Scientific) was dissolved in H_2_O to obtain a 100 mM stock solution. Drugs were stored in aliquots at -20°C. Treatments were performed with drugs or vehicle diluted in medium at the indicated concentrations as described in the Results. For qRT-PCR analysis, EU incorporation, and EdU incorporation, 30-50% confluent cells were treated for 24 h. For MTT analysis, cells were treated for 3-5 days refreshing the medium every 48 hours for the duration of the treatment.

### siRNA

40-60% confluent cells seeded on 24-well plates were transiently transfected with 10 nM Silencer Select pre-designed siRNAs (Thermo Fisher) targeting MYC (s9129+s9131), UBTF (s14613+s14615), RRN3 (s29324) or POLRMT (s10825+s10826) by using RNAi max Lipofectamine (Thermo Fisher) according to the manufacturer's instructions. As a negative control, cells were transfected with 10 nM siRNA Negative Control (Thermo Fisher). 24-48h after transfection, cells were analyzed by qRT-PCR or EdU incorporation.

### Western blot

Cells were lysed in RIPA Buffer (Cell Signaling) supplemented with 1 mM PMSF and EDTA-free Protease Inhibitor Cocktail (Roche). Total protein concentration was measured by using Coomassie Plus Protein Assay Reagent (Thermo Fisher). Equal amounts of proteins were separated by SDS PAGE electrophoresis and transferred onto a PVDF membrane according to standard procedures. Membranes were blocked with 5% non-fat dry milk diluted in TBS + 0.05% Tween 20 for 1 h at room temperature, incubated over night at 4°C with anti-MYC (C33) antibody (Santa Cruz Biotechnology) diluted in 5% milk, washed, incubated for 1 h at room temperature with HRP-conjugated anti-mouse antibody (GE Healthcare) diluted in 5% milk, washed, and incubated with Clarity Western ECL Substrate (Bio-Rad). Chemiluminescence was detected and quantified by using ChemiDoc Touch Imaging System (Bio-Rad).

### qRT-PCR

RNA was extracted with Trizol (Thermo Fisher), treated with DNase I (Thermo Fisher), and retrotranscribed by using High Capacity cDNA reverse transcription kit (Thermo Fisher). cDNA was analyzed by real time PCR by using the iQ SYBR Green Supermix (Bio-Rad) and primers amplifying MYC (sense: 5′-cctctcaacgacagcagct-3′, antisense: 5′-cagaaggtgatccagactctg-3′), pre-rRNA 5’ETS (sense: 5′-CTGAGGGAGCTCGTCGGTGT-3′, antisense: 5′-GCAGAGCGCCTCCGAAGTCA-3′), UBF (sense: 5′-ACCAAGCCACCTCCGAACAG-3′, antisense: 5′-AGGCAGGCTCTCGAGGAAAC-3′), RRN3 (sense: 5′-CGGAAACCTGAAAGAAGGTTTGC-3′, antisense: 5′-CTGGCGATTGTTCCTCTCAATG-3′), PTCD3-(5′-ACAACAGACTCCATGCTGATGT-3′, antisense: 5′-AAGCGAGGGTTCTATTCCAATG-3′), MRPS5 (sense: 5′-TGCCACAGGGCCATCATCAC-3′, antisense: 5′-CATGGAGGCCCTTCTTATCAG-3′), MRPS27 (sense: 5′-GGCTATGCACTTCTTGGGAAG-3′, antisense: 5′-GC ACATCGAGCGCTTCTCTAC-3′), TAF1C (5′-GGACA GGCTGCATTTCCAAGAG-3′, antisense: 5′-AGGTGA GGGCTGAGGCTGAT-3′), POLR1B (5′-TATGGAAG ATGCCATGATTGTGA-3′, antisense: 5′-TGTAATACG GATCTCCGTACTG-3′), POLR1E (sense: 5′-GGTGT GACTGCTCTGGTCAG-3′, antisense: 5′-GGTGCAAT GGCTGTTCTCCTC-3′), POLRMT (sense: 5′-CATCG AAAGGTGTCTGGAACAG-3′, antisense: 5′-CACGCC CATCCTTGGCATAC-3′), TFAM (sense: 5′-CATCTG TCTTGGCAAGTTGTCC-3′, antisense: 5′-ACTCCGC CCTATAAGCATCTTG-3′), mtRNA 12S (sense: 5′-GTGAGTTCACCCTCTAAATCAC-3′, antisense: 5′-GACTTGGGTTAATCGTGTGACC-3′), mtRNA 16S (sense: 5′-CCTGGTGATAGCTGGTTGTCC-3′, antisense: 5′-CAATTGGGTGTGAGGAGTTCAG-3′), mtRNA 12-16S (sense: 5′-GAGGAGACAAGTCGTAACATGG-3′, antisense: 5′-CTATATCTATTGCGCCAGGTTTC-3′), or GAPDH (sense: 5′-GAAGGTGAAGGTCGGAGTC-3′, antisense: 5′-GAAGATGGTGATGGGATTTC-3′) as we previously described [20]. Statistical significance was calculated by using the Student's t-test.

### Assessment of nucleolar EU incorporation

5-ethynyl uridine (EU) incorporation assay was performed by using Click-iT RNA Alexa Fluor Imaging Kit (Thermo Fisher). Subconfluent cells were incubated with 1 mM EU under standard growth conditions for 2 h, fixed with 4% paraformaldehyde for 10 min., permeabilized with PBS + 0.5 % Triton X100 for 15 min. and incubated with Click-iT reaction cocktail for 30 min., blocked with PBS+ (PBS plus 1% BSA, 1% FBS, 0.05% Tween 20) for 20 min, incubated with anti-B23 (Santa Cruz) diluted in PBS+ for 1 h at room temperature, washed four times with PBS+, incubated with anti-rabbit AlexaFluor 546 (Thermo Fisher) diluted in PBS+ for 1 h, washed four times with PBS, counterstained with 330 nM DAPI for 10 min., and mounted with Vectashield (Vector Laboratories). Cells were imaged with a fluorescence microscope using the same exposure time for each fluorescent channel. EU incorporation in the nucleolar compartment (identified by B23 staining) was assessed with Adobe Photoshop by multiplying the EU-positive nucleolar area (number of pixels) by the EU signal intensity (mean) in the B23-positive nucleolar compartment normalized to the EU signal intensity (mean) in the surrounding nucleoplasm. Statistical significance was calculated by Student's t-test.

### MTT assay

2-5 × 10^3^ cells/well were seeded in 24-well plates in four replicates, let attach overnight, and treated with the indicated drugs diluted in growth medium. After 3-5 days of treatment, cells were incubated for 2 hours under standard growth conditions with 0.5 mg/ml 3-(4,5-dimethylthiazol-2-yl)-2,5-diphenyltetrazolium bromide (MTT) (Sigma) and lysed with DMSO. Cell lysates were transferred into a 96- well plate, and absorbance at 570 nm was measured on a microplate spectrophotometer. Statistical significance was calculated by Student's t-test.

### EdU incorporation assay

EdU-incorporation was assessed with Click-iT EdU imaging kit (Thermo Fisher), according to the manufacturer's instructions. Briefly, cells were incubated under standard growth conditions with 10 μM EdU for 1h. After EdU incorporation, cells were fixed with 4% paraformaldehyde for 10 min., incubated with PBS plus 0.5 % Triton X100 for 10 min., washed with PBS plus 3% BSA, reacted with Click-iT reaction cocktail for 30 min., counterstained with 330 nM DAPI for 10 min, and mounted with Vectashield (Vector Laboratories). Cells were imaged with a fluorescence microscope. DAPI-positive cells and EdU-positive cells were counted in at least 5 random fields. Statistical significance was calculated by using the Student's t-test.

## SUPPLEMENTARY MATERIALS FIGURES


