# Supplementary material for: Undermining ribosomal RNA transcription in both the nucleolus and mitochondrion: an offbeat approach to target MYC-driven cancer
Source: Oncotarget. 2017 Dec 22;9(4):5016–31. doi: 10.18632/oncotarget.23579 (PMC5797030; doi:10.18632/oncotarget.23579)
Supplement: Supplementary file 1 [file oncotarget-09-5016-s001.pdf]

Undermining ribosomal RNA transcription in both the nucleolus and mitochondrion: an offbeat approach to target MYC-driven cancer

SUPPLEMENTARY MATERIALS

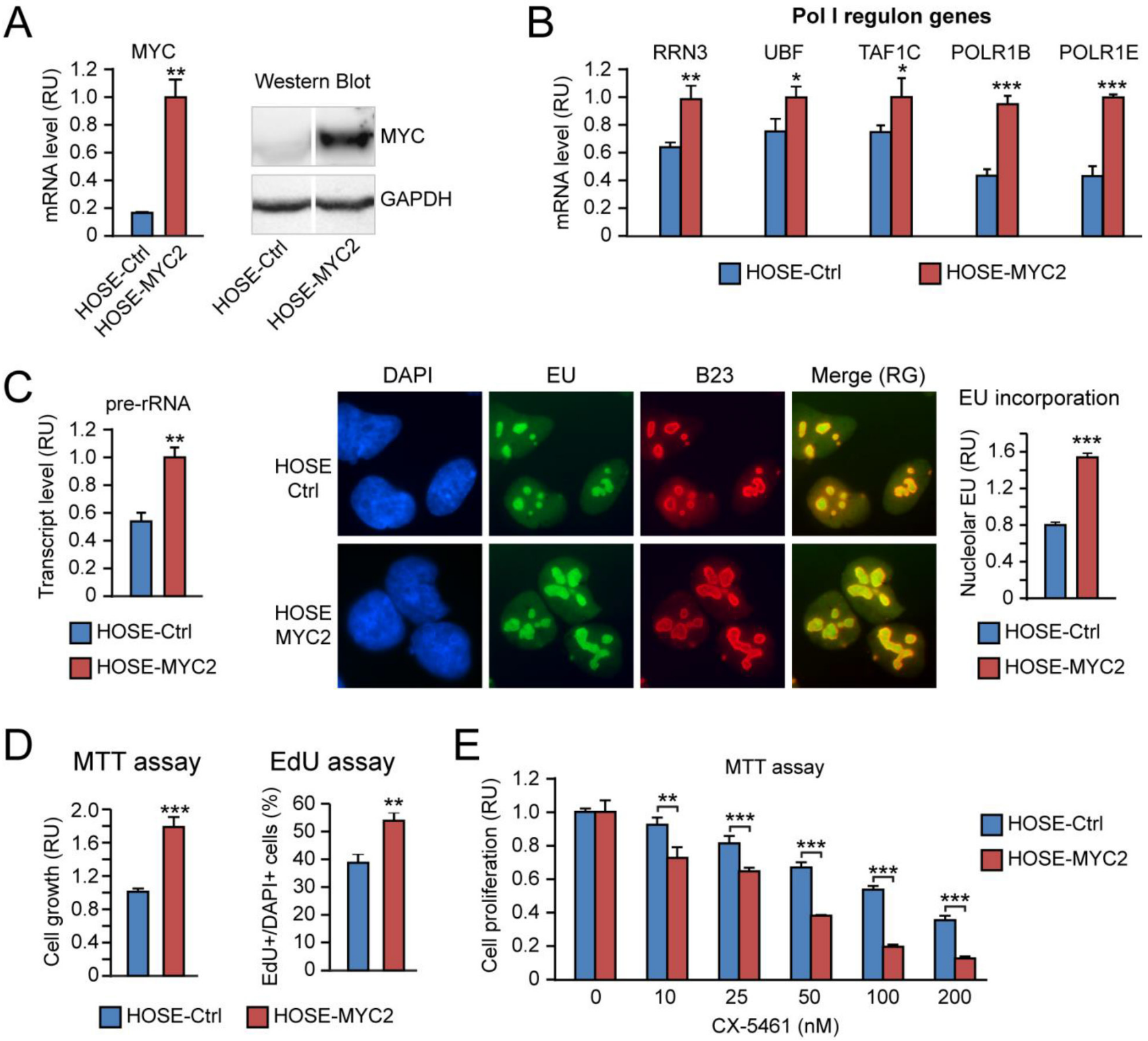

Supplementary Figure 1: Evidence in a different ovarian epithelial cell context that MYC overexpression, by increasing nucleolar rRNA transcription, sensitizes cells to the anti-proliferative action of the Pol I inhibitor CX-5461. **(A-B)** HOSE ovarian epithelial cells stably infected with exogenous MYC (HOSE-MYC2) display increased MYC level (see qRT-PCR in A, left, and Western Blot in A, right) and upregulation of Pol I regulon genes (see qRT-PCR in B) relative to HOSE cells carrying the cognate empty vector (HOSE-Ctrl). **(C)** Exogenous MYC expression in HOSE cells leads to upregulation of rRNA transcription, assessed both by pre-rRNA qRT-PCR (left) and quantification of EU incorporation into the B23-positive nucleolar compartment (middle and left). **(D-E)** HOSE-MYC2 cells display increased proliferation (see MTT assay in D, left, and EdU incorporation in D, right) and increased sensitivity to the anti-proliferative action of the Pol I inhibitor CX-5461 (E) relative to HOSE-Ctrl cells. $^{*}p<0.05$, $^{**}p<0.01$, $^{***}p<0.001$.

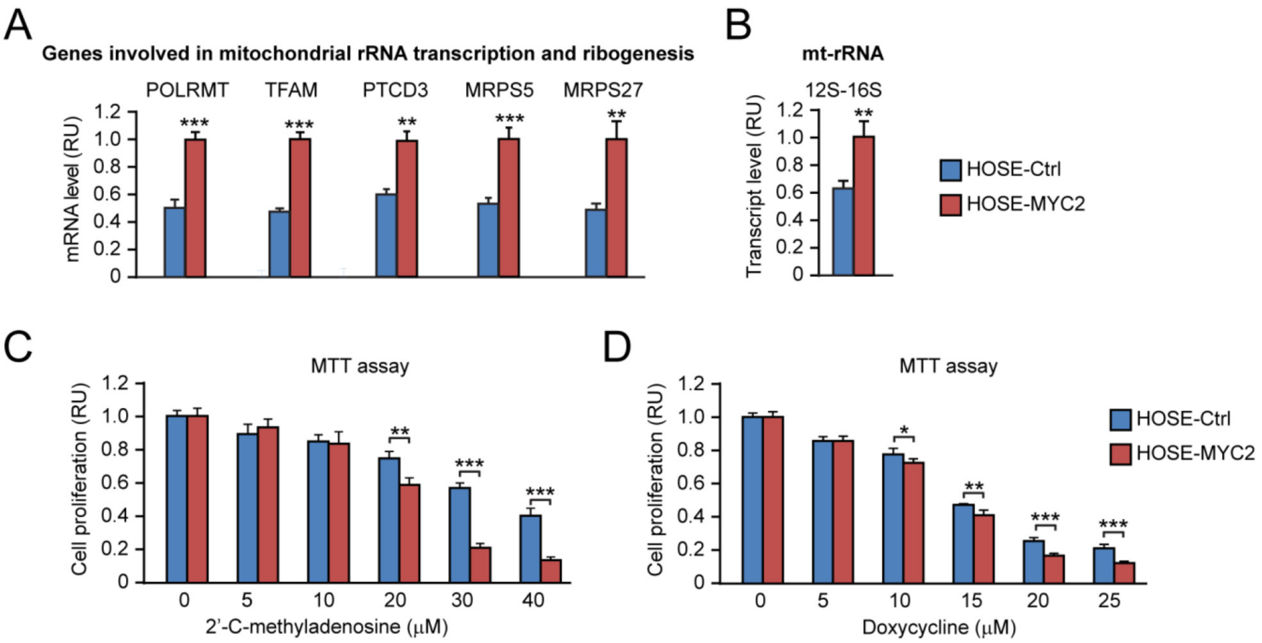

Supplementary Figure 2: Evidence that in the HOSE human ovarian epithelial cell context MYC overexpression, by increasing mt-rRNA transcription and mitoribogenesis, sensitizes cells to the anti-proliferative action of both 2'-C-MeA and doxycycline. **(A-B)** qRT-PCR showing that HOSE-MYC2 cells display upregulation of genes involved in mitochondrial rRNA transcription and ribogenesis (A) as well as increased pre-mtRNA 12-16S level (B) relative to HOSE-Ctrl cells. **(C-D)** Consistently, HOSE-MYC2 cells also display increased sensitivity to the anti-proliferative action of the POLRMT inhibitor 2'-C-MeA (C) as well as doxycycline, an inhibitor of mitoribosome function (D). $^{*}p<0.05$, $^{**}p<0.01$, $^{***}p<0.001$.
